# Synergy between HA cleavage site sequence and NA-mediated plasminogen recruitment as a virulence mechanism for low-pathogenic avian influenza

**DOI:** 10.1128/mbio.02466-25

**Published:** 2026-02-26

**Authors:** Hui Min Lee, Kate Sutton, William Harvey, Samantha Sives, Rute Maria Pinto, Eleanor Gaunt, Samantha Lycett, Sjaak de Wit, Lonneke Vervelde, Paul Digard

**Affiliations:** 1 The Roslin Institute, The University of Edinburgh, Easter Bush 3124, Midlothian, United Kingdom; 2 Royal GD, Deventer, the Netherlands; 3 Faculty of Veterinary Medicine, Utrecht University 8125, Utrecht, the Netherlands; Griffith University, Gold Coast, QLD, Australia

**Keywords:** influenza, neuraminidase, plasminogen, hemagglutinin, pathogenicity

## Abstract

An outbreak of H3N1 low-pathogenic avian influenza virus (LPAIV) in Belgium in 2019 caused unexpected levels of mortality and morbidity in poultry. These viruses possess an NA polymorphism associated with plasminogen (PLG) binding, as well as an atypical sequence around the HA cleavage site; accordingly, HA cleavage mediated by NA-driven PLG recruitment has been proposed to underlie their systemic spread and pathogenicity. To test this, we established a reverse genetics system for A/chicken/Belgium/460/2019 and created single mutations in HA (K345R) and NA (S122N) that restored the viruses to normal consensus, as well as an HA/NA double mutant. Confirming previous work, trypsin-independent spread and HA cleavage of wild-type Ck/Belgium were observed in the presence of fetal bovine serum containing PLG *in vitro*. Dose-dependent HA cleavage and trypsin-independent spread were also observed in the presence of purified chicken PLG. Compared to the wild-type virus, both HA cleavage and virus spread *in vitro* were reduced by the HA K345R mutation and further blocked by the NA mutation S122N. PLG-mediated HA cleavage was seen in a variety of avian cell lines and chicken organoids, excluding cell type-dependent effects. Furthermore, *in ovo* tests showed that mutant viruses unable to recruit PLG were less able to replicate systemically in chicken embryos. Bioinformatics analyses revealed other viruses that could potentially recruit PLG, including two independent outbreaks of H6N1 viruses, one of which we confirmed PLG-driven spread *in vitro*. We conclude that PLG recruitment by NA is a general virulence mechanism of N1 LPAIVs.

**IMPORTANCE:**

Avian influenza viruses are divided into high or low pathogenicity based on the sequence of their hemagglutinin (HA) and their lethality in chickens. The majority of AIV strains circulating in the wild are of low pathogenicity both in waterfowl and when they spill over into domestic poultry. However, some low-pathogenicity strains can cause serious disease in poultry. A severe 2019 outbreak of an H3N1 strain has been suggested to result from the viral neuraminidase (NA) recruiting cellular plasminogen to proteolytically activate HA. Here, we confirmed that the sequence of the NA at position 122 is the primary determinant of plasminogen-driven HA cleavage, but that the unusual sequence at the HA cleavage also contributes to pathogenicity. Furthermore, we show that this N1 NA sequence motif can be used to identify other unexpectedly virulent AIV strains. This work, therefore, adds to our ability to risk assess AIV strains from sequence-based surveillance.

## INTRODUCTION

Avian influenza viruses (AIV) are classified into high-pathogenic (HPAI) or low-pathogenic (LPAI) viruses based on their hemagglutinin (HA) subtype and sequence near the HA cleavage site. Almost without exception, HPAI viruses (HPAIVs) are H5 or H7 subtypes that possess a polybasic cleavage site (PBCS), whereas LPAI viruses are all other subtypes and H5 or H7 subtypes without a PBCS (1, 2). In gallinaceous poultry such as chickens and turkeys, HPAIVs show systemic spread and rapidly fatal disease, whereas infection by LPAI viruses is largely restricted to the respiratory tract and intestinal organs and often causes few or no clinical signs (3). However, not all LPAI viruses are phenotypically low pathogenic; certain strains can be highly virulent, spread systemically in the animals, and lead to an overt outbreak (4–10).

In 2019, an LPAI H3N1 outbreak involving various poultry species started in Belgium and spread regionally for 4 months, including to three farms in France (9). The outbreak was notable for its association with dramatic drops in egg production, tropism for the reproductive tract, and high mortality in chickens (9). Our laboratory study using an H3N1 isolate from the outbreak, A/chicken/Belgium/460/2019 (Ck/Belgium), showed 58% mortality and 100% drop in egg production in 34-week-old specific-pathogen-free (SPF) laying hens (8). The virus was also detected in the oviducts of the hens at 9–10 days post-infection (8). This indicated that the H3N1 strain was indeed a virulent LPAIV capable of systemic spread in poultry. Therefore, it was of interest to identify the molecular reasons that contribute to the increased virulence of Ck/Belgium, both to understand the reasons for it and to enhance risk prediction of future novel AIV strains as they arise.

The surface of an influenza virus particle contains two glycoproteins: HA and neuraminidase (NA). In order to activate virus infectivity and permit cell entry, the HA precursor polypeptide (HA0) must be cleaved by host proteases into two functional subunits, HA1 and HA2 (11–13). Without this specific proteolysis event freeing the N-terminus of the HA fusion peptide, viral and host membranes cannot be fused, and the virus entry fails. Therefore, HA sequence around the HA cleavage site can be an important determinant of virus pathogenicity in different hosts (2, 14). The PBCS in HPAIV HAs are cleaved intracellularly by ubiquitously expressed furin family proteases (15, 16), whereas the HAs from LPAI viruses with a monobasic cleavage site are usually activated extracellularly by secreted trypsin-like proteases found in the airway or gut lumens, but not within the internal organs (12, 17). An additional mechanism for HA cleavage, which depends on the sequence of the viral NA, has been found in certain strains of influenza A virus. In a human-isolated but mouse-adapted H1N1 strain, A/Wilson Smith Neurotropic/1933 (WSN), the presence of a C-terminal lysine and loss of glycosylation site at position 130 (WSN numbering) on NA leads to NA binding to plasminogen (PLG), which is subsequently converted to plasmin that cleaves adjacent HA molecules (18, 19). PLG recruitment by NA in WSN has been associated with increased pathogenicity in mice, including neurotropism, presumably because of the systemic availability of PLG (18). Consistent with this, PLG-knockout mice infected with another laboratory-adapted H1N1 strain, A/Puerto Rico/8/1934 (PR8), which is also able to use PLG for HA cleavage, showed higher survival and lower weight loss compared to wild-type animals (20).

**Fig 1:**
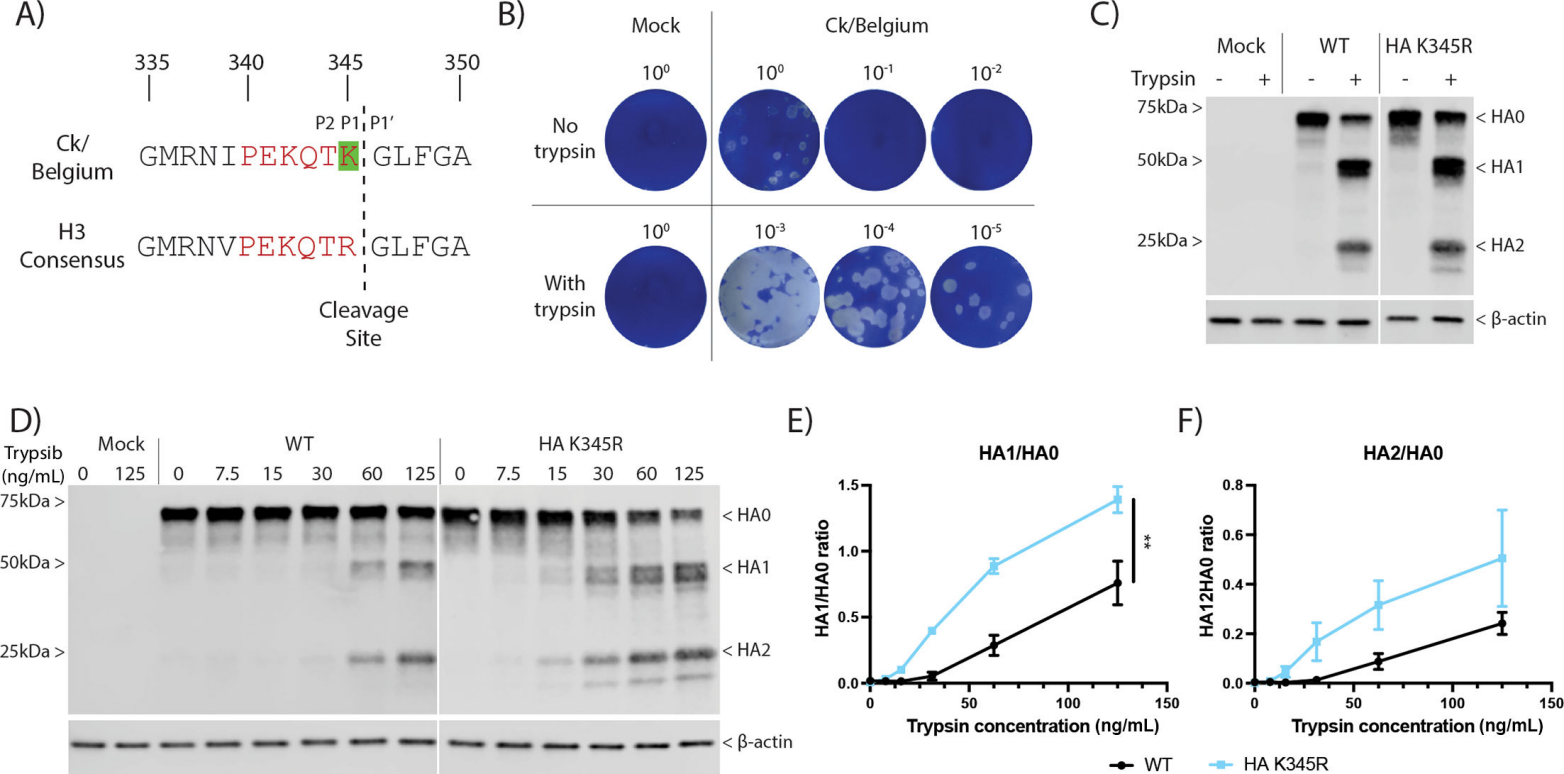
Trypsin dependency of CK/Belgium in cell culture. (**A**) Alignment of HA sequences near the cleavage site for Ck/Belgium and an H3 consensus. Red lettering indicates the cleavage site motif, while the amino acid highlighted in green at position 345 indicates the polymorphism of Ck/Belgium in this motif compared to the H3 consensus. (**B**) Plaque titration in MDCK cells (with trypsin) of supernatant collected from mock or Ck/Belgium-infected CLEC213 cells (MOI = 0.001) incubated in the presence or absence of trypsin for 48 h. (**C**) CLEC213 cells were infected with WT or HA K345R at an MOI of 3 for 16 h, and 1 μg/mL trypsin was added for the last 2 h of infection. Cell lysates were examined by SDS-PAGE and western blotting for HA and β-actin. Positions of molecular mass markers are indicated on the left. (**D**) CLEC213 cells were infected with WT or HA K345R viruses at an MOI of 3 for 16 h, and the indicated trypsin concentrations were added for the last 2 h of infection. SDS-PAGE and western blotting were performed to detect HA and β-actin. Positions of molecular mass markers are shown on the left. (**E and F**) Ratios of cleaved (HA1 or HA2) to uncleaved HA (HA0) products were quantified by densitometry, and data are pooled from two independent infection and transfection experiments, expressed as mean ± SEM. An unpaired *t*-test of area under the curve for each condition was used for statistical analyses. **P < 0.01.

Notably, the NA from the H3N1 Ck/Belgium outbreak also lacks the equivalent glycosylation site (at position 122) to the WSN NA, and a previous study has shown that another isolate from the outbreak, A/chicken/Belgium/1940/2019, exhibited trypsin-independent spread in cell culture in the presence of PLG-containing serum (21). Furthermore, virus replication was blocked by a plasmin inhibitor, 6-aminohexanoic acid (6-AHA), suggesting a mechanism for the increased virulence of the Ck/Belgium family viruses (21). In addition, the Ck/Belgium HA also contains an atypical monobasic cleavage sequence, with a lysine (K) at the HA1 boundary (P1 position), instead of the more common arginine (R) residue seen in H3 HAs (Fig. 1A). A previous study using WSN has reported that a non-consensus amino acid at the P2 position of the HA cleavage site affected virus virulence under PLG-dependent conditions (22), indicating the possibility of collaboration between the atypical HA cleavage site and PLG binding by NA (21, 22). Accordingly, we set out to test the molecular hypotheses that the unexpectedly high virulence of Ck/Belgium lineage viruses resulted from PLG recruitment by NA working in concert with a modified HA monobasic cleavage site. We confirmed PLG-mediated cleavage of the Ck/Belgium HA in the context of virus infection and found that the atypical NA and HA sequences combine for efficient PLG-mediated spread in a variety of avian cell systems. We also identified PLG-mediated spread in a separate H6N1 outbreak, suggesting that this may be a general pathogenicity mechanism for LPAIV virulence.

## MATERIALS AND METHODS

### Cells

Chicken ANP32A-expressing Madin-Darby canine kidney cells (MDCK/ANP32A; a kind gift from Prof Massimo Palmarini, University of Glasgow Center for Virus Research, UK), quail fibroblast cells (QT-35; a kind gift from Dr Laurence Tiley, University of Cambridge, UK) (23), and human embryonic kidney cells (HEK293T; ATCC) were cultured in Dulbecco’s modified Eagle’s medium (DMEM; Sigma) supplemented with 10% fetal bovine serum (FBS; Gibco), 100 U/mL penicillin, and 100 µg/mL streptomycin (Gibco). In total, 1 µg/mL puromycin was added for the selection marker in MDCK/ANP32A cells. Chicken lung epithelial cells (CLEC213; a kind gift from Dr. Sascha Trapp, French National Institute for Agriculture, Food and Environment, France) (24) were cultured in DMEM-F12 (Sigma) supplemented with 8% FBS, 100 U/mL penicillin, and 100 µg/mL streptomycin. Chicken embryonic fibroblast cells (DF-1) (25) and duck embryonic fibroblast cells (CCL-141; a kind gift from Dr. Leah Goulding, University of Nottingham, UK) were cultured in DMEM-F12 supplemented with 10% FBS, 100 U/mL penicillin, and 100 µg/mL streptomycin.

### Antibodies and antisera

The following primary antibodies were used: polyclonal rabbit anti-H3 for A/HKx31 (H3N2; a kind gift from Prof Janet Daly, University of Nottingham, UK), laboratory-made polyclonal rabbit anti-nucleoprotein (NP; 2915) (26), polyclonal goat anti-influenza A H1N1 antiserum (5315-0064, Bio-Rad), polyclonal rabbit anti-plasminogen (PA5-34677, Invitrogen), monoclonal rat anti-α-tubulin (clone YL1/2, Invitrogen), and monoclonal mouse anti-ß-actin (ab8226, Abcam). Secondary antibodies used were: Alexa Fluor 800-conjugated goat anti-rabbit IgG (Invitrogen), Alexa Fluor 680-conjugated goat anti-rat IgG (Invitrogen), IRDye 594RD goat anti-rabbit IgG (LI-COR Biosciences), and IRDye 680RD donkey anti-mouse IgG (LI-COR Biosciences).

### Viruses and reverse genetics

H3N1 A/chicken/Belgium/460/2019 (Ck/Belgium) (8) and H3N2 A/Udorn/307/1972 (Udorn; a kind gift of Dr. Richard Compans, Emory University, Atlanta) (27) have been previously described. H1N1 A/Wilson Smith Neurotropic/1933 (WSN) and H6N1 A/chicken/Netherlands/917/2010 were sourced from the Division of Virology, Department of Pathology, University of Cambridge, and Royal GD virus strain collections, respectively.

To establish a reverse genetics system for Ck/Belgium, individual full-length segments corresponding to an in-house sequence of the coding regions of A/chicken/Belgium/460/2019 clone GD4 (hereafter Ck/Belgium) with the missing untranslated regions obtained by deriving an H3N1 consensus sequence were ordered from Genewiz, UK, and subcloned into a bi-directional RNA polymerase I/II “pDUAL” reverse genetics vector (28) at the *BsmBI* restriction site. Each segment was sequenced to confirm identity. Desired mutations were introduced using a QuikChange Lightning Site-Directed Mutagenesis kit (Agilent) according to the manufacturer’s instructions. Wild-type Ck/Belgium and its mutant derivatives were then produced by reverse genetics as previously described (29). Briefly, HEK293T cells were transfected with eight pDUAL plasmids encoding each segment of influenza virus. Following overnight incubation, the medium was replaced with serum-free DMEM supplemented with 0.14% bovine serum albumin (BSA; Merck) and 1 μg/mL L-(tosylamido-2-phenyl) ethyl chloromethyl ketone (TPCK)-treated trypsin (Worthington Biochemicals) and co-cultured with MDCK/ANP32A cells to obtain efficient rescue. The cell culture supernatant was collected at 3 days post-transfection and clarified before being used to infect 10-day-old embryonated chicken eggs. Eggs were chilled overnight at 2 days post-infection, and the allantoic fluid was harvested, clarified, and titrated by plaque assays on MDCK/ANP32A cells. The presence or absence of specific mutations, as well as the receptor binding site of the HA, was confirmed by sequencing.

All reverse genetics work used a loss-of-function approach and was carried out under a license (GMRA1811) from the UK Health & Safety Executive. All work with avian viruses was carried out in a segregated BSL2 laboratory in the Roslin Institute, separate from where mammalian strains of influenza A virus are handled.

### Plaque assay

Confluent cell monolayers of MDCK/ANP32A cells were used to measure virus titer. Cells were infected with 10-fold serial dilutions of virus for an hour at 37°C and then overlaid with serum-free DMEM containing 0.14% BSA, 1 μg/mL TPCK-treated trypsin, and 0.8% Avicel (Dupont). Following 3 days of incubation at 37°C, the cells were fixed with neutral-buffered formalin for 20 min and stained with 0.1% toluidine blue (Sigma) for another 20 min.

### Immunofluorescence

Cells were infected with viruses at the MOIs stated in the figure legends and incubated in DMEM-F12 in the absence or presence of 10% FBS for 24 or 72 h. Cells were then fixed with phosphate-buffered saline (PBS)/4% formaldehyde (Thermo Fisher Scientific) for 20 min, washed three times with PBS, blocked for 30 min with 1% FBS in PBS, and incubated with primary antibody for an hour. Following 3 PBS/1% FBS washes, cells were incubated with species-specific secondary antibody for an hour and subsequently Hoechst dye (to stain nuclei) incubation for 10 min. Cells were washed three times with PBS before mounting with ProLong Gold antifade reagent (Invitrogen) and visualized using a DMRB fluorescence microscope (Leica).

### Immunoblotting

Cells were infected with viruses or transfected with plasmids and then incubated in the absence or presence of 10% FBS, 6-AHA (Sigma), or purified chicken PLG (Abcam) according to experimental design. To measure the kinetics of HA cleavage, cells were treated with 50 μg/mL cycloheximide (Sigma) before the addition of PLG for varying lengths of time. Cells were then lysed in 2× homemade Laemmli buffer (30) and separated by 4%–20% SDS-PAGE (Bio-Rad). Proteins were transferred onto nitrocellulose membranes (Thermo Fisher Scientific) using a Transblot Turbo semi-dry transfer system (Bio-Rad). Membranes were blocked with Intercept blocking buffer (Li-COR Biosciences) for 1 h before overnight incubation with primary antibodies at 4°C. Following three washes in PBS/0.1% Tween 20, membranes were incubated with species-specific secondary antibodies for 1 h. Membranes were washed three times in PBS/0.1% Tween 20 and visualized using an Odyssey Fc imager (Li-COR Biosciences). Densitometric analyses were performed using Image Studio Lite Software (Li-COR Biosciences).

### Flow cytometry

Cells were infected with viruses at the MOIs stated in the figure legends and incubated in the absence or presence of chicken PLG according to experimental design. Cells were fixed with PBS/4% paraformaldehyde for 20 min, washed three times in PBS, and incubated with primary antibody diluted in cell staining buffer (Biolegend) for 1 h. Cells were washed three times in cell staining buffer and incubated with species-specific secondary antibody for 1 h. After three PBS washes, the percentage of virus-infected cells was determined using an LSR Fortessa X-20 flow cytometer (BD Biosciences); 10,000 events were recorded by gating on live single cells. Data analysis was performed using FlowJo (BD Biosciences).

### Fluorescent labeling of PLG

Purified chicken PLG was labeled with Alexa Fluor 488 (AF488) using an AF488 Microscale Protein Labeling Kit (Invitrogen). To confirm protein recovery and labeling, 1,000, 500, 250, and 125 ng of AF488-conjugated PLG were separated by 4%–20% SDS-PAGE, followed by Coomassie Brilliant Blue R-250 stain (Bio-Rad) according to the manufacturer’s protocol and visualization under white light or fluorescence detection at 488 nm using an Odyssey M imager (Li-COR Biosciences). To examine NA-PLG binding, cells were infected with viruses at an MOI of 3 for 14 h, followed by treatment with or without 50 ng/mL AF488-PLG for 2 h. Cells were then fixed with PBS/4% paraformaldehyde for 20 min, washed three times in PBS, and the percentage of PLG-positive cells was quantified by flow cytometry.

### Chicken embryo pathogenesis model

Pathogenesis of Ck/Belgium was determined in chicken embryos as previously described (31). Briefly, embryonated chicken eggs incubated for 10 days (Embryonic Day 10; National Avian Research Facility, The Roslin Institute) were inoculated via the allantoic cavity route with 100 pfu of virus diluted in 100 μL of serum-free medium. To test the effects of 6-AHA in this system, eggs were inoculated in the allantoic cavity with 10 mg 6-AHA in 100 μL of serum-free medium. At 2 days post-infection, embryos were killed by chilling and membrane disruption or membrane disruption and decapitation. Decapitated embryos were washed twice in PBS and fixed for several days in 10% neutral buffered formalin. Five embryos per virus were mounted onto paraffin wax. Embryo tissues were sectioned onto slides and stained with hematoxylin and eosin (H&E) by the Easter Bush Pathology Service. Further unstained sections were used to determine the presence of viral NP by immunohistofluorescence. Deparaffinization and rehydration, followed by heat-induced antigen retrieval using sodium citrate buffer (10 mM sodium citrate, 0.05% Tween 20, pH 6.0), were performed on tissue sections before staining with primary antibody overnight at 4°C, followed by three washes in PBS the next day. Tissue sections were incubated with species-specific secondary antibody and Hoechst for an hour at room temperature. After three PBS washes, sections were mounted using ProLong Gold antifade reagent (Invitrogen), scanned using a NanoZoomer XR instrument (Hamamatsu), and analyzed using NDP view, version 2.3, software (Hamamatsu).

### Generation and infection of chicken 3D intestinal organoids

Embryonic Day 18 Hy-Line Brown Layer eggs were sourced from the National Avian Research Facility, University of Edinburgh, UK. Duodenum, jejunum, and ileum were extracted from chicken embryos and placed in PBS (Mg^2+^ and Ca^2+^ free) until further processing. For each independent culture, intestines from 3 to 4 embryos were pooled. Intestinal villi were isolated as described (32). In brief, intestinal tissues were longitudinally opened and cut into 3 mm pieces, followed by enzymatic digestion (Clostridium histolyticum Type I Collagenase, 0.2 mg/mL Merck) for 50 min at 37°C, 200 rpm. Villi were collected using a 70-µm cell strainer (Corning) by rinsing the inverted strainer, followed by centrifugation at 107 × *g* for 4 min. Organoids were seeded in Petri dishes with 12 mL of Floating Organoid Media (FOM), consisting of Advanced DMEM/F12 supplemented with 1× B27 Plus, 10 mM HEPES, 2 mM L-Glutamine, and 50 U/mL Penicillin/Streptomycin. Organoids were cultured at 37°C with 5% CO_2_.

On day 3 of culture, 200 organoids were seeded in 24-well plates (Corning) in 400 µL of FOM supplemented with or without 50 ng/mL of chicken PLG. Organoids were mock-infected or infected with 1,000 pfu of viruses according to experimental design, with five wells infected per time point. At 1, 24, and 48 h post-infection, organoids were collected by centrifugation at 10,000 × *g* for 2 min, lysed using QIAGEN RLT buffer supplemented with 10 µg/mL β−2-mercaptoethanol (Sigma-Aldrich), and stored at −20°C until use. Cell culture supernatants were collected, frozen on dry ice, and stored at −80°C until use.

### RNA isolation, reverse transcription, and qPCR

RNA was isolated using QIAGEN mini-RNA kits according to the manufacturer’s instructions. Reverse transcription was performed using the Superscript III First Strand Synthesis System (18080051, Thermo Fisher Scientific) according to the manufacturer’s instructions using random hexamers and 100 ng of total RNA. The cDNA samples were stored at −20°C until use. To measure mRNA levels, a 1:5 dilution of cDNA was mixed with 10 µL of ABI TaqMan Gene Expression Master Mix (4369016, Applied Biosystems), 1 µL of 20× EvaGreen (31000, Biotum, VWR-Bie & Berntsen), and specific primer pairs for AIV segment 7 and chicken GAPDH (forward and reverse) at a final concentration of 1.15 μM. Reactions were performed in triplicate, and no template controls without cDNA were included to detect any potential non-specific amplification. The efficiency of each reaction was calculated based on serial 10-fold dilutions of a calibration sample. The qPCR reaction was carried out at 50°C for 2 min, 95°C for 10 min, followed by 40 cycles at 95°C for 15 s and 60°C for 1 min.

Primers used were as follows: for AIV segment 7, forward CTTCTAACCGAGGTCGAAACGTA and reverse CACTGGGCACGGTGAGC (33); for GAPDH, forward GAAGGCTGGGGCTCATCTG and reverse CAGTTGGTGGTGCACGATG (accession number AF047874).

### Analysis of gene expression via 96.96 IFC Fluidigm Dynamic Array qPCR

cDNA was synthesized from 200 ng of RNA using a high-capacity reverse transcription kit (Applied Biosystems) according to the manufacturer’s instructions. cDNA was pre-amplified with TaqMan Pre-Amp Master Mix (Applied Biosystems) and 2.5 µL of a 200 nM mixed pool of primer pairs (as described previously [34]; see Table S1 for the gene list used here) at 95°C for 10 min, followed by 14 cycles of 95°C for 15 s and 60°C for 4 min. Pre-amplified cDNA was treated with 16 U/µL Exonuclease I (New England Biolabs) at 37°C for 30 min before heat inactivation at 80°C for 15 min. Pre-amplified, exonuclease-treated cDNA was analyzed through qPCR with the microfluidic 96.96 Dynamic Fluidigm array on the BioMark HD instrument (BioMark). Assay mixes were prepared with 2.25 µL 2× assay loading reagent (Fluidigm), 2.5 µL of primer pair mix (1.15 µM), and 0.25 µL low EDTA TE buffer. Sample mixes were prepared with 2.5 µL TaqMan Gene Expression Master Mix (Thermo Scientific), 0.25 µL 20× EvaGreen DNA binding dye (Biotum), 0.25 µL 20× GE Sample Loading reagent (Fluidigm), and 2 µL of pre-amplified, Exonuclease-treated cDNA. Thermal cycling conditions were: 50°C for 2 min, 70°C for 30 min, 25°C for 10 min, 50°C for 2 min, 95°C for 10 min, and then 30 cycles of 95°C for 15 s, 60°C for 1 min, and fluorescence emission was recorded after each cycling step. Raw quantitation cycle (Cq) data were collated with the Real-Time PCR Analysis software v 3.1.3 (Fluidigm), setting parameters of a quality threshold (0.65), baseline correction (derivative), and Cq threshold method to auto (global). Raw Cq values were processed with GenEx7 (MultiD), with correction for primer efficiency and reference gene normalization. Stability of expression of reference genes: TATA box binding protein (TBP), Tubulin alpha chain (TUBA8B), beta-actin (ACTB), beta-glucuronidase (GUSB), glyceraldehyde-3-phosphate dehydrogenase (GAPDH), and ribosomal 28S (r28S) were evaluated with NormFinder (GenEx7). The geometric mean of the most stable (ACTB, r28S, and TBP) was used for normalization. The ratios of virus-infected samples against mock-infected samples were then calculated using the normalized data and further compared between the control and PLG-treated group.

### Sequence analyses

All N1 NA amino acid sequences from viruses of HA subtype H2–H13 and all non-human H1N1 were downloaded from GISAID (35). Human H1N1 sequences were excluded to make the analysis more computationally tractable. These 46,199 sequences were aligned using MAFFT v7.511 using default settings (36, 37). With the resulting alignment, apparent amino acid insertions present in fewer than 5% of sequences were trimmed. Sequences in the resulting trimmed alignment with amino acid coverage below 90% were excluded, leaving 45,001 sequences. The amino acid triplets at positions 130–132 (WSN numbering) were identified. Sequences without complete amino acid information at these positions (*n* = 13) were excluded. Retained sequences were classified as possessing an N-glycosylation motif, Asn-X-Ser/Thr (N-X-S/T), where X is any amino acid except proline (Pro/P), at positions 130–132, or not. Sequences lacking an N-glycosylation motif and without an amino acid other than lysine (Lys/K) at the C-terminal position 453 were defined as potential PLG binders. To place natural sequences possessing this proposed PLG-binding site in the context of the wider diversity of N1 NA proteins, a clustering analysis was used. Within each HA subtype, pairwise amino acid distances were computed from aligned protein sequences, and clusters were identified using hierarchical clustering with the nearest chain algorithm (38). Among all sequences lacking the PLG-binding motif, one sequence was randomly selected from each cluster with at least five representatives. This resulted in 119 sequences that were combined with 74 natural sequences possessing a PLG-binding motif for phylogenetic reconstruction. Phylogeny was reconstructed from aligned protein sequences using RAxML-NG (39) with rates of amino acid substitution estimated using the FLU model (40). The best-scoring maximum likelihood tree was midpoint rooted and plotted in R using the ggtree (41).

### Statistical analyses and image processing

All graphs were plotted, and all statistical analyses were performed using GraphPad Prism version 10. Individual tests used depended on the data under analysis and are given in figure legends. Image data were subjected to linear adjustments of brightness and contrast and color balance to aid visualization using LicoR Image Studio (for blots) or Adobe Photoshop (for microscopy images). Figures were prepared using Adobe Illustrator.

## RESULTS

### The unusual Ck/Belgium HA cleavage site reduces susceptibility to trypsin cleavage

To understand the molecular factors contributing to the increased pathogenicity of Ck/Belgium, the HA sequence of Ck/Belgium was compared with the consensus HA sequence of all avian H3 viruses submitted to NCBI from January 2005 to December 2024. This highlighted that Ck/Belgium has a lysine (Fig. 1A; highlighted in green) just before the cleavage site instead of an arginine commonly found in H3 viruses. First, we determined if this unusual sequence motif conferred a pseudo-HPAIV-like ability of Ck/Belgium to replicate in cells in the absence of trypsin. Chicken lung epithelial CLEC213 cells were infected at low MOI with Ck/Belgium and incubated in serum-free medium with or without trypsin. Subsequent plaque titration in MDCK cells (in the presence of trypsin) showed that Ck/Belgium only replicated in the presence of exogenous protease (Fig. 1B). To further understand the effect of the unusual HA cleavage site, we created a Ck/Belgium HA K345R mutant, which had the H3 consensus sequence restored and then directly assayed HA cleavage by infecting cells with wild type (WT) and mutant virus, followed by western blotting for HA. This showed that both WT and HA K345R needed trypsin for cleavage in CLEC213 cells, as only uncleaved HA0 precursor was visible in the absence of trypsin, while a mix of HA0 and cleaved HA1 and HA2 were present with trypsin (Fig. 1C). To provide a more quantitative measurement of trypsin susceptibility, cells were infected with both viruses, then treated with a range of trypsin concentrations and assessed for HA cleavage. From the western blot (Fig. 1D), HA K345R showed higher susceptibility to trypsin cleavage than the WT. To examine this in the absence of other viral proteins, cells were transfected with the plasmids encoding WT or mutant HAs and subjected to the same trypsin titration and western blot analysis. Again, the HA K345R mutant showed greater trypsin cleavability (Fig. S1). Densitometric measurement of the HA1/HA0 and HA2/HA0 ratios from infection and transfection data confirmed the higher sensitivity of the HA mutant to trypsin (Fig. 1E and F), although this was only statistically significant for HA1/HA0 ratios. Thus, the unusual HA sequence of Ck/Belgium does affect trypsin susceptibility, but by reducing cleavability rather than enhancing it.

### The unusual NA and HA sequence polymorphisms in Ck/Belgium affect PLG cleavage of HA

**Fig 2:**
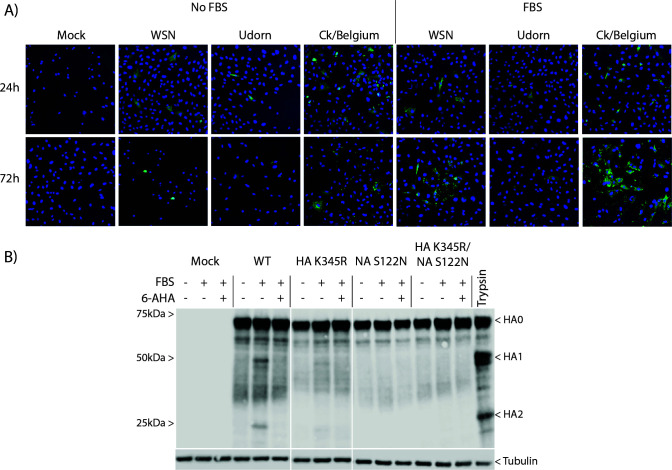
Effects of serum on spread and HA cleavage of Ck/Belgium. (**A**) CLEC213 cells were infected with WSN, Udorn, or Ck/Belgium at an MOI of 0.001 and incubated in the absence or presence of 10% FBS. Cells were fixed and stained with anti-H3 (Udorn and Ck/Belgium) or anti-H1 (WSN) (both in green) and Hoechst (blue) and visualized by fluorescent microscopy. Images for 24 h and 72 h post-infection were taken with a 20× magnification lens. (**B**) CLEC213 cells were infected with WT or mutant viruses at an MOI of 3 and incubated in the absence or presence of 10% FBS and 500 μg/mL 6-AHA as indicated. A positive control well was incubated with 1 μg/mL trypsin to provide markers for HA1 and HA2. Cell lysates were harvested 16 h post-infection and examined by SDS-PAGE and western blotting for HA and tubulin. Positions of molecular mass markers are shown on the left. Images are representative of 2–3 independent experiments.

A previous study using another virus isolate from the same Belgium outbreak (A/chicken/Belgium/1940/2019) identified the loss of a glycosylation site at amino acid position 122 on NA (position 130 in WSN numbering), which, by analogy with WSN, would allow NA binding of PLG and subsequent trypsin-independent spread (21). Our Ck/Belgium isolate shares the same potential PLG-binding motif in its NA; hence, we tested whether it too exhibited trypsin-independent spread in the presence of FBS containing PLG. CLEC213 cells were infected with Ck/Belgium, WSN (as a positive control), and Udorn (as a negative control) at low MOI and incubated in the absence or presence of FBS for 24 or 72 h before staining the cultures with anti-HA to identify infected cells. Individual infected cells were observed for all viruses at 24 h, with or without FBS (Fig. 2A). This pattern of single infected cells remained at 72 h for all viruses without serum and Udorn with serum. However, clear microplaques were visible at 72 h for both WSN and Ck/Belgium in the presence of FBS, indicating virus spread, thus corroborating the findings of Schon and colleagues (21).

In order to test the hypothesis that the identity of the amino acid at position 122 on Ck/Belgium NA confers the ability of the virus to spread in the absence of trypsin, as well as to test for any phenotypic association with the unusual HA cleavage site, we created two additional mutants: an NA S122N mutant (restoring the potential glycosylation site) and an HA/NA double mutant, HA K345R/NA S122N. We then infected CLEC213 cells with WT or mutant viruses at high MOI in the absence or presence of FBS and a plasmin-specific inhibitor, 6-AHA, before examining HA cleavage by western blot of cell lysates. Low levels of HA cleavage were observed with the WT virus in the presence of serum, but not in the absence of FBS, nor with FBS further treated with plasmin inhibitor (Fig. 2B). The HA K345R mutant showed reduced HA cleavage in the presence of FBS than the WT virus but was still inhibited by 6-AHA, while the NA S122N and HA K345R/NA S122N viruses did not show detectable HA cleavage under any conditions. These results support the hypothesis that the Ck/Belgium NA recruits PLG for HA cleavage and that the unusual HA cleavage site contributes to the efficiency of this process.

**Fig 3:**
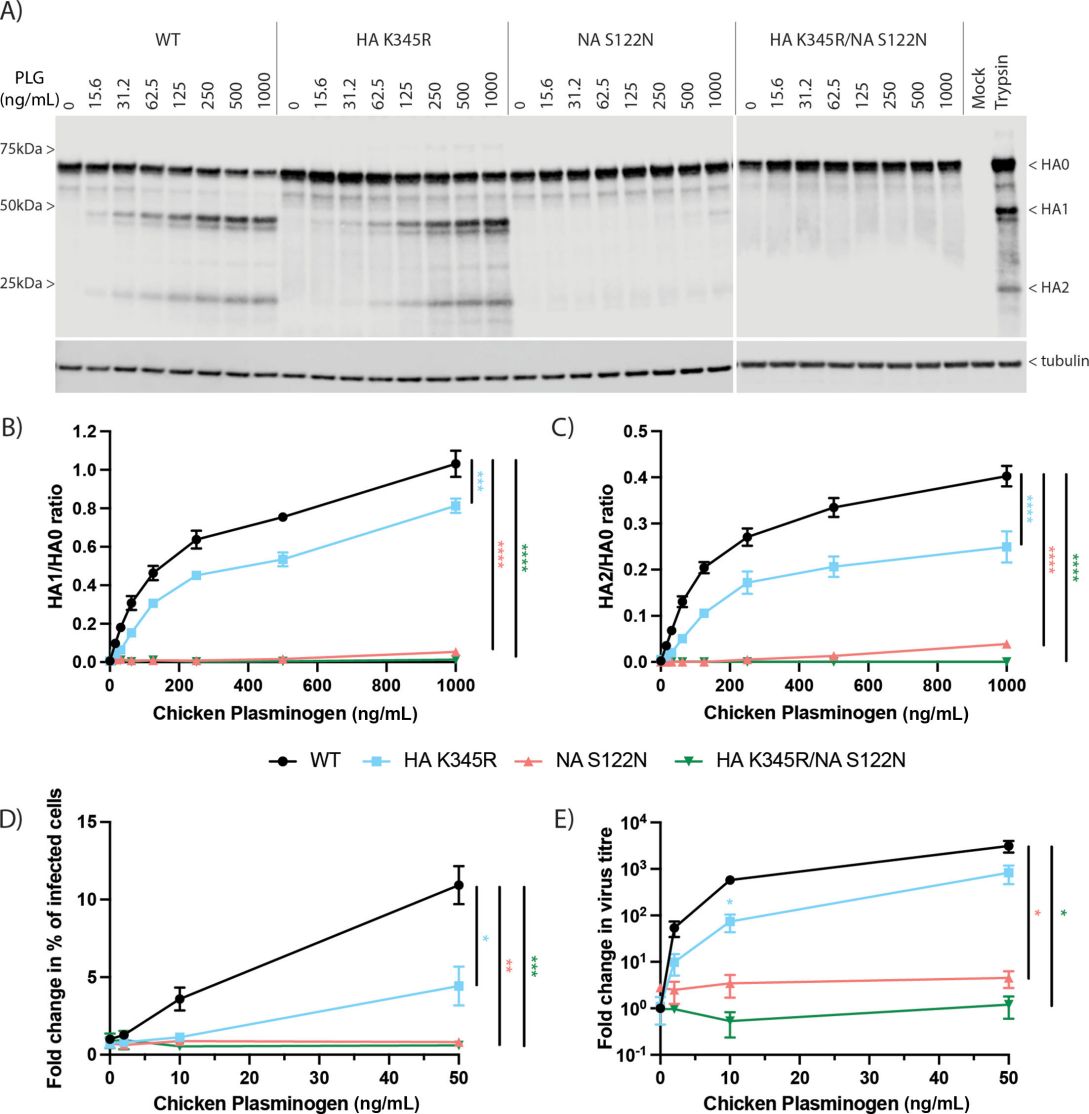
Synergy between the Ck/Belgium HA cleavage site and NA PLG recruitment motif. (**A–C**) CLEC213 cells were infected with WT or mutant viruses at an MOI of 3 and incubated in the presence of a range of chicken PLG concentrations for 16 h. (**A**) Cell lysates were examined by SDS-PAGE and western blotting for HA and tubulin. Positions of molecular mass markers are shown on the left. (**B and C**) Ratios of cleaved to uncleaved HA products from replicate blots were quantified by densitometry. Data are mean ± SEM of three independent experiments. (**D and E**) CLEC213 cells were infected with WT or mutant viruses at an MOI of 0.001 and incubated with the indicated amounts of PLG for 24 h. (**D**) Cells were fixed and stained with anti-H3, and the number of infected cells was quantified by flow cytometry, while (**E**) released infectious virus was titrated by plaque assay. Data are presented as fold increase induced by PLG relative to WT virus without PLG, and values are mean ± SEM of 3–4 independent experiments. Statistical analyses are the area under the curve for each virus (**B–E**) or one-way ANOVA and Dunnett’s multiple comparisons test for HA K345R in (**E**).

To confirm that HA cleavage resulted from the presence of PLG and not another 6-AHA-sensitive factor present in FBS, we next tested the effects of adding host species-matched purified chicken PLG during virus infections. CLEC213 cells were infected with WT or mutant viruses at high MOI and treated with a range of chicken PLG concentrations (working substantially below the plasma concentrations measured for chickens and humans [42–44]); then, HA cleavage was determined at 16 h by western blotting as before. Chicken PLG caused dose-dependent HA cleavage for WT and HA K345R, whereas the HA1/HA2 cleavage products of the NA S122N mutant virus were only faintly observed with 1 μg/mL of PLG (the highest concentration used; Fig. 3A). No HA cleavage was detected for the double mutant HA K345R/NA S122N. Quantification of HA1/HA0 (Fig. 3B) and HA2/HA0 (Fig. 3C) ratios from replicate experiments showed that the HA K345R mutation significantly reduced cleavage compared to the WT HA, as well as confirming only residual cleavage with the NA mutant and no detectable cleavage for the double mutant.

To further probe the effect of the HA PBCS site on PLG-mediated cleavage, we measured the kinetics of HA processing. Cells were infected at high MOI and left until 14 h post-infection to allow cell-surface HA to accumulate. Further translation was then blocked by the addition of cycloheximide, and chicken PLG was added to the culture supernatant to allow HA cleavage to proceed. Analysis of HA processing by western blot showed that WT HA was cleaved at a significantly (*P* < 0.05) higher rate than HA K345R (Fig. S2).

In the WSN strain of IAV, PLG is recruited to the cell surface by binding to the surface-exposed C-terminal lysine of NA, unmasked by the loss of a glycosylation site (18, 19). To examine this mechanism for our H3N1 system, we fluorescently labeled the purified chicken PLG (Fig. S3A through C) and then tested whether this bound to the surface of cells infected with WT or NA S122N viruses. CLEC213 cells infected with the WT virus bound significantly more PLG than either cells infected with the NA S122N virus or mock-infected cells (Fig. S3D), confirming the importance of the altered NA glycosylation site in the avian virus system.

To examine the effect of PLG on infectious virus production, CLEC213 cells were infected at low MOI and incubated with a range of PLG concentrations. After 24 h, the number of infected cells was measured by flow cytometry after fixing and staining for surface H3, while the amount of infectious virus released was measured by plaque titration. The spread of the WT virus showed a dose-dependent response to PLG concentration and was significantly higher than all the mutants, particularly viruses with the NA mutation (Fig. 3D). Titers of infectious virus also increased with PLG concentration, and again, all mutations reduced virus replication (Fig. 3E). These results indicate that the unusual Ck/Belgium HA cleavage site and NA recruitment of PLG collaborate to induce HA cleavage and virus spread *in vitro*.

### Synergy between the HA cleavage site and NA-mediated PLG recruitment is not cell type-dependent and occurs *ex vivo* and *in ovo*

**Fig 4:**
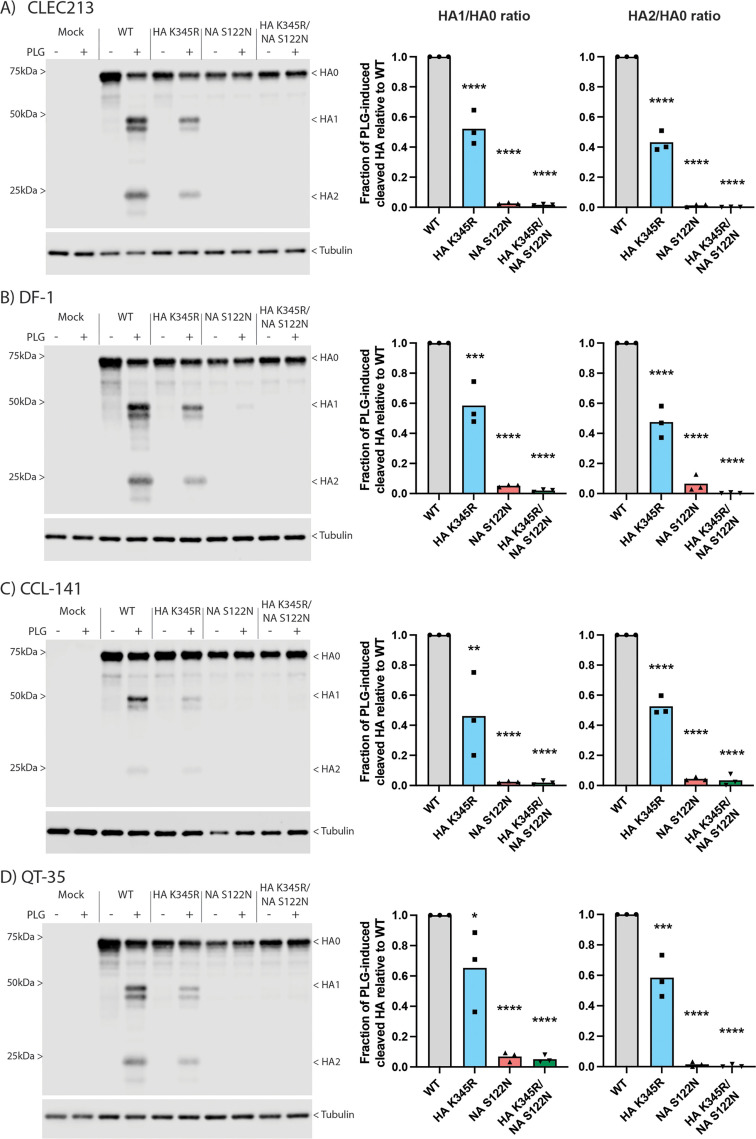
The effects of Ck/Belgium HA cleavage site and NA PLG-binding motifs are not cell type or species-specific. PLG-induced HA cleavage was determined in chicken (**A and B**), duck (**C**), and quail (**D**) cell lines. Cells were infected with WT or mutant viruses at an MOI of 3 for 16 h in the absence or presence of 250 ng/mL PLG before analysis by western blot for HA and tubulin. Images are representative of three independent experiments. Dots on graphs represent densitometric ratios of HA1/HA0 and HA2/HA0 from individual experiments, normalized to the respective WT values. Bars indicate the means of three independent experiments. One-way ANOVA followed by Dunnett’s multiple comparisons test was performed for statistical analyses. *P < 0.05, **P < 0.01, ***P < 0.001, ****P < 0.0001.

PLG activation is a tightly regulated process with multiple cell-surface stimulatory and inhibitory factors, whose abundance can vary by tissue or cell type (44). All our experiments so far were performed in CLEC213 cells; hence, it was therefore important to test the significance of the Ck/Belgium HA and NA sequence motifs in other avian cells. Using a single concentration of 250 ng/mL PLG, cleavage of WT Ck/Belgium HA was again significantly higher than HA K345R, and no cleavage was found for NA S122N and HA K345R/NA S122N in infected CLEC213 cells (Fig. 4A). Very similar results were obtained using another chicken cell line (DF-1 fibroblasts; Fig. 4B), as well as in duck (CCL-141; Fig. 4C) and quail (QT-35; Fig. 4D) fibroblast cell lines. Thus, the HA processing induced by the collaborative effects of the unusual HA cleavage site and NA-recruited PLG was not specific to a single type of chicken cell but also applied to immortalized cells from other avian species.

**Fig 5:**
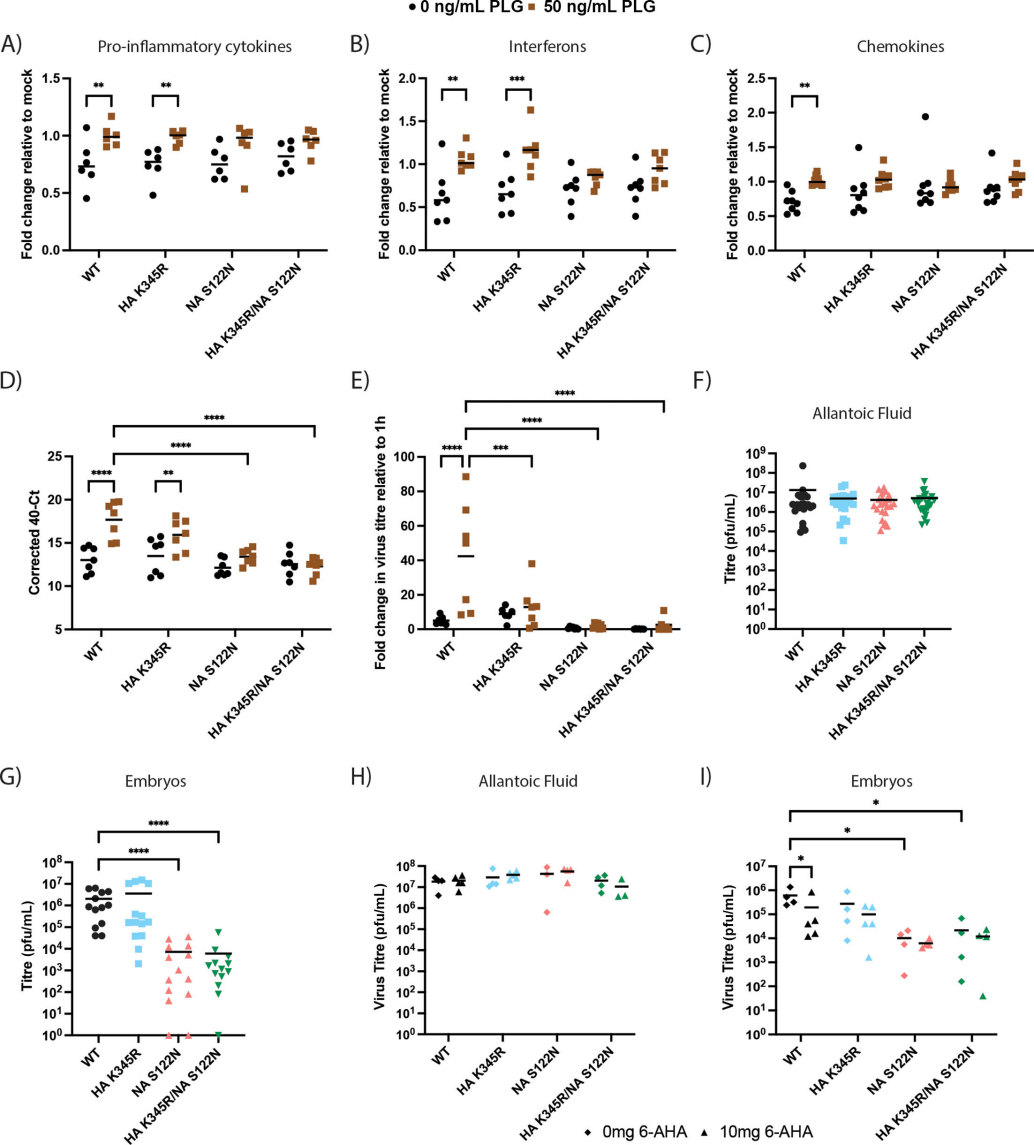
The effects of Ck/Belgium HA cleavage site and NA PLG-binding motifs on host cell responses and in *ex vivo* and *in ovo* systems. (**A–C**) CLEC213 cells were infected (or mock infected) with WT or mutant viruses at an MOI of 3 and incubated in the presence or absence of 50 ng/mL PLG for 16 h. Total cellular RNA was then extracted, and a chip-based Fluidigm RT-qPCR assay was performed to measure the expression of various immune-related genes in the categories of (**A**) pro-inflammatory cytokines, (**B**) interferons, or (**C**) chemokines. Each dot represents the fold change of an immune gene relative to the mock-infected cells, and lines represent the median. The data are the means of two independent experiments. Two-way ANOVA followed by Sidak’s multiple comparison test was performed for statistical analyses. (**D and E**) Chicken organoids were infected with 1,000 pfu of WT or mutant viruses in the absence or presence of 50 ng/mL PLG for 24 h. Virus replication was measured by (**D**) qPCR of the intracellular segment seven or (**E**) plaque assay of the released virus. Each dot represents a pool of organoids derived from 3 to 4 chicken embryos, and lines represent the means of 6-7 organoid pools from two independent experiments. Two-way ANOVA (qPCR) or mixed-effects analysis (plaque assay), followed by Dunnett’s multiple comparison test, was performed for statistical analyses. (**F–I**) The allantoic cavities of 10-day-old embryonated chicken eggs were inoculated with 100 pfu of WT or mutant viruses and incubated for 2 days. Virus titers in (**F**) allantoic fluid and (**G**) chicken embryos were examined. Chicken eggs were also inoculated with 10 mg 6-AHA during virus infection, and the effects of 6-AHA on the virus titers in (**H**) allantoic fluid, and (**I**) chicken embryos were measured. Each dot represents virus titers from an individual egg, and lines indicate the means. Statistical differences were determined using the Kruskal-Wallis test followed by Dunn’s multiple comparisons test for F and G or two-way ANOVA followed by Tukey’s multiple comparisons test for **H** and **I**. *P < 0.05, **P < 0.01, ***P < 0.001, ****P < 0.0001.

In order to further examine the biological relevance of PLG-mediated HA cleavage, we infected CLEC213 cells with WT or mutant viruses at an MOI of 3 for 16 h and measured the expression of various immune response-related genes using a chip-based RT-qPCR “Fluidigm” assay specific for chicken genes (34, 45). The addition of PLG to CLEC213 cells infected with WT virus significantly increased the expression of a repertoire (Table S1) of pro-inflammatory cytokines, interferons, and chemokines, whereas neither virus with NA S122N showed such a response (Fig. 5A through C). Altering only the HA cleavage site had less effect. Next, we used chicken organoids as a complex cell model of infection in primary cells (32). Groups of approximately 200 organoids pooled from 3 to 4 chick embryos were infected with WT or mutant viruses in the absence or presence of PLG. Virus replication was examined 24 h later by quantifying the intracellular expression of segment 7 RNA as well as virus released into the supernatant. The addition of PLG increased segment 7 accumulation significantly in WT and HA K345R infections, while the NA S122N mutation suppressed this PLG-induced increase, both alone and with the HA mutation (Fig. 5D). Although variable between batches of organoids, the addition of PLG also significantly increased the titer of released WT, but not mutant viruses (Fig. 5E). Overall, these data further corroborate the importance of the HA cleavage site and NA PLG-binding motifs for Ck/Belgium replication.

**Fig 6:**
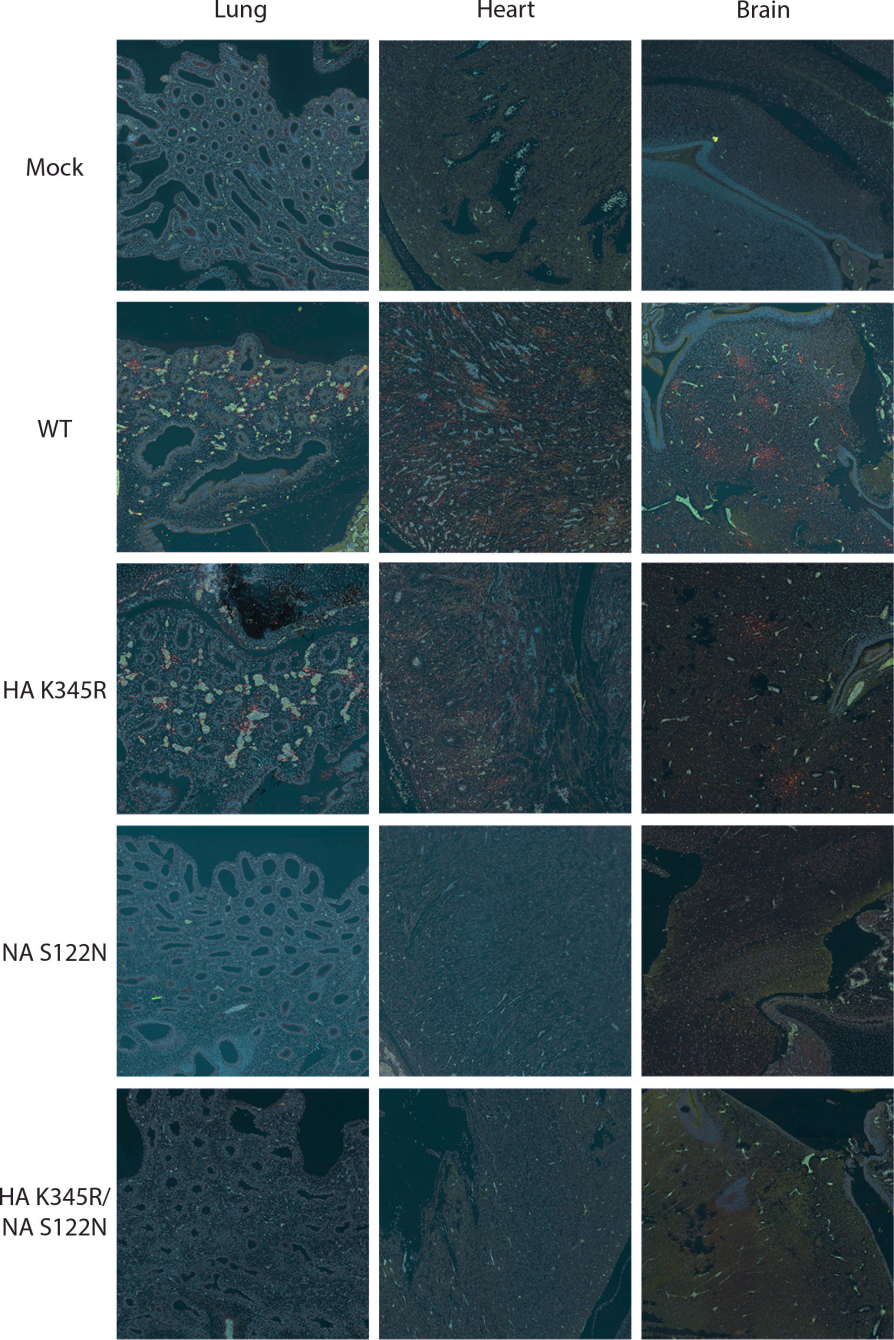
The Ck/Belgium NA PLG-binding motif facilitates systemic replication in the chick embryo model**.** The allantoic cavities of 10-day-old embryonated chicken eggs were inoculated with 100 pfu of WT or mutant viruses and incubated for 2 days. Embryos were fixed, sections cut, and then immunofluorescently stained for viral NP and cellular nuclei using Hoechst dye. Images show the lung, heart, and brain of chicken embryos infected with the indicated viruses and are representative of 10 embryos per condition. Red, NP; blue, nucleus; green, autofluorescence.

As a further *in ovo* model of infection, we inoculated the allantoic cavity of 10-day-old embryonated chicken eggs with WT and mutant Ck/Belgium viruses. There were no differences in the titers of the various viruses harvested from the allantoic fluid 48 h later (Fig. 5F). WT Ck/Belgium and the HA K345R mutant also replicated well in the chicken embryos, but in contrast, NA S122N and HA K345R/NA S122N showed notably lower virus titers (Fig. 5G). To test if the differences in virus replication in chicken embryos could be linked to PLG activity, we treated the chicken eggs with 6-AHA. While no difference was observed in the virus titers in allantoic fluid with or without 6-AHA (Fig. 5H), chicken embryos infected with WT virus again showed notably higher titers than viruses with the NA S122N mutation in the absence of 6-AHA (Fig. 5I). The addition of 6-AHA significantly reduced the titers of WT but not the mutant viruses, suggesting a PLG-specific effect *in ovo*. These results were also supported by immunostaining of the chicken embryos for virus antigen. In lung, heart, and brain sections, NP expression was only observed for WT and HA K345R viruses, whereas the staining for NA S122N and HA K345R/NA S122N was similar to that of mock-infected embryos (Fig. 6). These findings indicated the importance of NA-mediated PLG recruitment in the pathogenesis of Ck/Belgium.

### PLG recruitment by N1 NA has also evolved in moderately virulent H6 subtype LPAIVs

**Fig 7:**
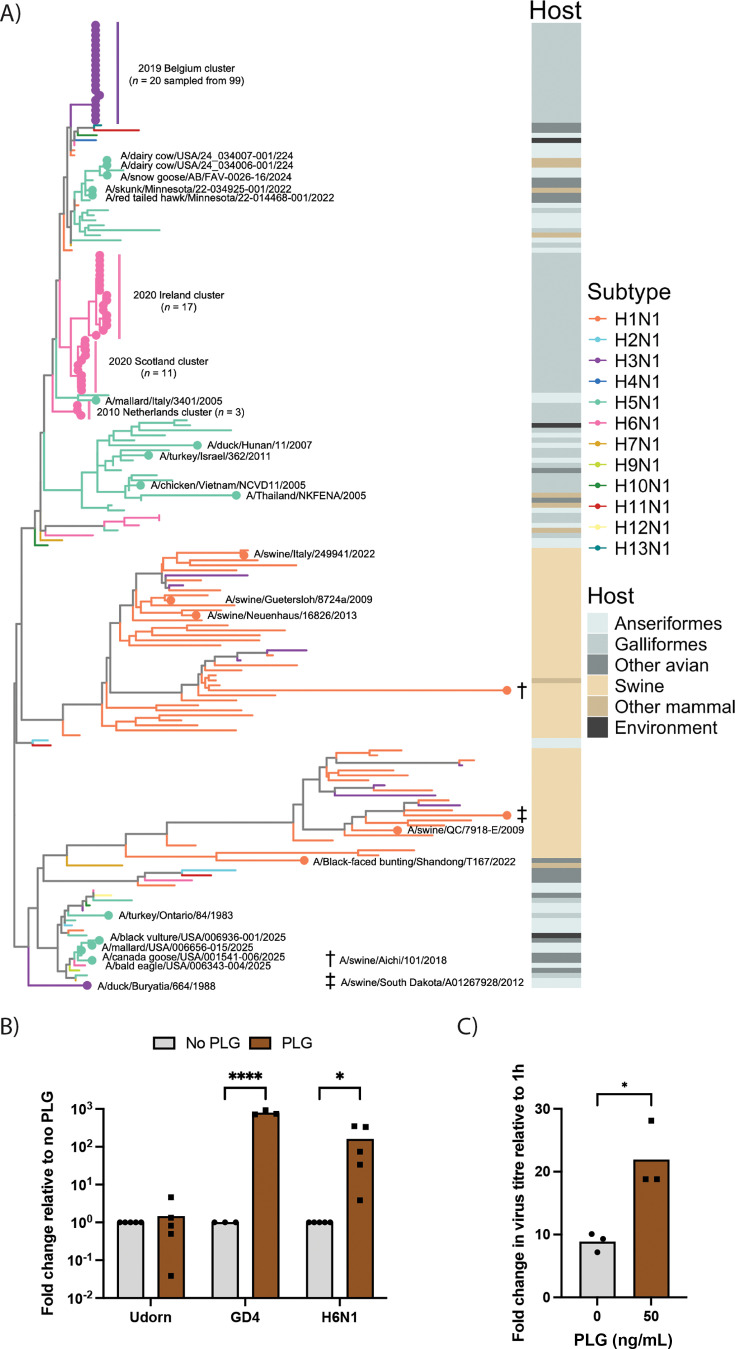
Identification of potential NA-mediated PLG recruitment in other N1 subtype viruses**.** (**A**) NA phylogeny was reconstructed from amino acid sequences of 74 N1 NAs with a PLG-binding motif, and 119 other sequences representing the diversity of N1 NAs. The tree is mid-point rooted, and branches are colored by subtype. The positions of viruses with PLG-binding motifs are highlighted by circles; virus names or epidemiological clusters are labeled. Alongside, a colored column indicates host type, which includes the avian orders Anseriformes (ducks, geese, and swans) and Charadriiformes (gulls, waders, and auks). (**B**) Experimental tests of PLG utilization by a 2010 Netherlands H6N1 outbreak virus. CLEC213 cells were infected with Udorn, Ck/Belgium, or H6N1 viruses at MOI = 0.001 for 48 h in the absence or presence of 50 ng/mL PLG. (**C**) Chicken organoids were infected with H6N1 virus in the absence or presence of 50 ng/mL PLG for 24 h. Virus replication in (**B**) and (**C**) was examined by plaque assay of released virus. Dots represent biological replicates in (**B**), or pools of organoids from 3 to 4 chicken embryos in (**C**), and bars indicate the means. Statistical differences were determined using 2-way ANOVA, followed by Sidak’s multiple comparisons test in (**B**) and an unpaired *t*-test in (**C**). *P < 0.05, ****P < 0.0001.

Schon and colleagues previously searched the Influenza Research Database for other N1 NA sequences that contained a PLG-binding motif, identifying 15 independent virus isolates not linked to the Belgian 2019 H3N1 outbreak (21). Of these, the largest category (discounting multiple sequences of lab-adapted WSN, PR8, and a reassorted derivative of the latter) was human infections, while only two were independent isolates of LPAIVs, both from the 1980s. To further investigate the emergence of the PLG-binding motif across N1 NAs, we carried out a similar search using the Global Initiative on Sharing All Influenza Data (GISAID) database (35). From over 38,000 NA sequences that met inclusion criteria (we excluded human seasonal viruses for computational tractability), we found 165 that lacked the key glycosylation site and had a C-terminal lysine, which may permit PLG binding. Of these, 153 were inferred to be from individual naturally occurring virus isolates (Tables S1 and S2). These potential PLG-utilizing viruses span a date range from 1983 to 2025, possess H1, H3, H5, or H6 subtype HAs, and come from a range of host species, both mammalian and avian, but predominantly domestic poultry (Table 1). To gain an understanding of the distribution of these PLG-binding viruses among the wider diversity of N1 NAs, they were placed in a phylogeny representative of protein diversity. Figure 7A highlights NAs with PLG-binding motifs, as well as indicating subtype and the associated host types across the tree. Most (100% or 83.3%) were H3N1 sequences, with the vast majority belonging to the 2019 Belgium outbreak (*n* = 99, of which 20 are plotted in Fig. 7A) and one other sequence (A/duck/Buryatia/664/1998; identified in a previous study [21]) being highly diverged from this outbreak. Outside of H3N1 strains, the next largest group of NAs possessing the PLG-binding motif was H6N1 viruses (31 sequences). We noted two clusters of H6N1 viruses in which the motif indicative of PLG-binding appears to have emerged independently. Based on the dates and countries from which the isolates were collected, they derive from two epizootic clusters: three sequences representing a 2010 outbreak in the Netherlands (4), while the other 28 sequences are from linked but genetically distinct outbreaks in Ireland, Northern Ireland, and Scotland (5). All four outbreaks were associated with notable drops in egg production and increased mortality. For the Ireland/UK outbreak, there was also evidence of systemic virus spread within the birds (5). We also found H5N1 and H1N1 subtype viruses (15 and 7 sequences, respectively) that possessed the PLG-binding motif. These tend to occur as singletons in the tree, suggesting independent evolution, except for two clusters of viruses sampled as part of the ongoing epizootic of HPAI H5N1 in the USA (Table 1). However, closer examination of genotypes and NA sequences involved suggested that these also represented several independent emergence events. The majority of other H5N1 strains with potential PLG-recruiting NAs are also HPAIVs. Thus, the N1 PLG-binding motif has evolved independently on multiple occasions, but most notably in LPAIV outbreaks in poultry, in the context of virus strains exhibiting increased pathogenicity.

**TABLE 1:** Categories of non-laboratory adapted influenza A virus N1 strains known or suspected to recruit PLG

Grouping	HA subtype	LPAI/ HPAI	H5 HA clade^*a*^	GenoFlu genotype	Host species	No. of sequences^*b*^	Date range	Atypical HA CS	HA CS motif^*c*^
Sporadic swine	H1	n/a			Swine	6	2009-2018	1/6	PS(I/V)QSR
Ck/Belgium	H3	LPAI			Domestic poultry	99	2019	yes	PEKQT(R/K)
Sporadic H5 Gs/Gd lineage	H5	HPAI	1, 2.3.4, 2.2.1.1a		Various	4^*d*^	2005-2011	n/a^*e*^	n/a^*e*^
		HPAI	2.3.4.4b (Eurasian lineage NA)	B2.1, B1.3, B3.6, B3.13	Various	5	2022-2024	n/a^*e*^	n/a^*e*^
		HPAI	2.3.4.4b (American lineage NA)	D1.1	Wild birds	4	2025	n/a^*e*^	n/a^*e*^
Ck/Netherlands	H6	LPAI			Domestic poultry	3	2010	no	PQIETR
Ck/Ireland (ROI/NI)	H6	LPAI			Domestic poultry	17	2020	no	PQIETR
Ck/GB	H6	LPAI			Domestic poultry	11	2020	no	PQIETR
Sporadic LPAIV	H1/H3/H5	LPAI			Various avian	4	1983- 2022	no	n/a^*e*^

^*a*^ For H5 HA clade 2.3.4.4b, viruses are distinguished as possessing either neuraminidase of either Eurasian or American AIV lineages.

^*b*^ Number of natural viruses with PLG-binding motifs identified in analysis of 44,988 N1 sequences (10,695 H1N1, 69 H2N1, 310 H3N1, 36 H4N1, 32,629 H5N1, 727 H6N1, 2 H8N1, 48 H9N1, 90 H10N1, 63 H12N1, and 6 H13N1).

^*c*^ Consensus sequences at the C-terminal end of HA1 are given for the particular subtype; residues in brackets indicate variability (where present) with the second amino acid being the atypical sequence.

^*d*^ One isolate lacks an associated HA sequence.

^*e*^ Virus lineages where the HA cleavage sites are either generally variable (HPAIVs) and/or too few to define a consensus are indicated as n/a (not applicable).

We had an isolate of the 2010 Netherlands H6N1 outbreak available (A/chicken/Netherlands/917/2010). Accordingly, to determine if this virus could utilize PLG, CLEC213 cells were infected at low MOI with Udorn, Ck/Belgium, or the H6N1 LPAI viruses and incubated without trypsin but in the absence or presence of PLG for 48 h. Plaque titration of released virus showed that addition of PLG significantly increased the virus titers of Ck/Belgium and H6N1, but not Udorn (Fig. 6B). Furthermore, PLG addition also significantly increased replication of the H6N1 virus in chicken organoids (Fig. 6C). We conclude that similarly to Ck/Belgium, the atypical N1 sequence of the Netherlands H6N1 outbreak also leads to PLG-mediated spread of the virus, potentially explaining the higher than normal pathogenicity of the outbreak viruses.

## DISCUSSION

Despite being categorized as an LPAI, H3N1 viruses from the Ck/Belgium family have been characterized as moderately virulent in poultry, showing systemic replication as well as causing appreciable mortality and a severe drop in egg production (8). The unexpected pathogenicity of the outbreak strain has been linked to PLG-cleavage of the HA, potentially mediated by loss of a glycosylation site in NA that, by analogy with the laboratory-adapted WSN and PR8 strains, leads to recruitment of PLG to the cell surface by NA (18, 21, 46). Here, we report molecular tests of this hypothesis. We confirmed PLG-driven HA cleavage and virus spread of Ck/Belgium using purified chicken PLG in avian cell systems from three species, including fibroblast, epithelial, and organoids. Using reverse genetics, we show that the key determinants of this are indeed the loss of a potential glycosylation site in NA, along with a variant monobasic cleavage site in the H3 HA that potentiates PLG cleavage and increases virus replication and cytokine expression *in vitro*, as well as *ex vivo* and *in ovo* systems. Furthermore, we identify further outbreaks of unexpectedly pathogenic H6N1 LPAIVs with similar traits, suggesting that this is a general virulence mechanism for N1 LPAIVs.

This study brings the number of virus lineages experimentally verified to use PLG recruitment by an N1 NA for HA processing to four: the H1N1 WSN and PR8 families, the 2019 Belgium H3N1 outbreak, and the 2010 Netherlands H6N1 LPAIV outbreak. WSN (18, 19, 22) and now the Belgian H3N1 viruses have been shown to depend primarily on the N1 mutation that destroys a potential N-linked glycosylation site and also possess a further adaptive mutation in the HA cleavage site that enhances plasmin-mediated proteolysis. Although both sequence motifs are important, the NA change is clearly more consequential, as its reversal to normal consensus has a greater effect on replication *in vitro* and pathogenicity *in ovo* than restoring the HA cleavage site to the more usual sequence [(18, 19, 22) and this study]. Furthermore, in a separate study, we found that the NA S122N mutant virus substantially attenuated virulence and reduced tissue tropism in both pullets and adult laying hens (47), confirming the importance of this sequence polymorphism for pathogenicity. Outside the context of the H3N1 outbreak, the NA PLG-binding polymorphism was sufficient to correctly identify the Netherlands H6N1 outbreak virus as using PLG, although its HA has the usual H6 PQIETR/GLF cleavage site sequence (Table 1). The viruses from the linked 2020 outbreaks of moderately virulent H6N1 LPAIVs in Irish and UK poultry flocks (4, 5) also have a normal HA consensus cleavage site, but nevertheless, we think it is plausible that they, too, will prove to be able to use PLG to mature HA.

Whether the acquisition of an unusual HA cleavage site sequence represents the “fine-tuning” of the PLG recruitment virulence mechanism or is selected in some but not all virus lineages for other reasons (perhaps HA subtype-specific) remains to be determined. The unusual cleavage site in WSN did not obviously alter its ability to be cleaved by trypsin (22), but the Ck/Belgium HA showed a moderate reduction in trypsin-cleavability. In mammals, PLG is both broadly expressed and secreted from the blood into other tissues, including the lung and central nervous system (48, 49), but it is unclear whether a virus that can use PLG only uses PLG, or (more plausibly perhaps), HA is simply cleaved by whatever protease with the right specificity comes along first. If the latter is true, then it would be interesting to test whether the Ck/Belgium HA cut site also affects cleavage by airway proteases and thus represents a balancing act between transmissibility and systemic replication.

Further relating to the diversity of proteases involved in HA cleavage is the intriguing number of H5 HPAIV HAs, primarily from the A/Goose/Guandong/1/1996 lineage, which have an N1 NA without the key glycosylation site (Table 1). At first sight, it seems unlikely that a virus that has evolved a highly efficient furin-cleavable HA would gain an advantage from being able to also use PLG. Furin-like proteases that cleave HA are usually viewed as ubiquitous in cell types and tissues, explaining the systemic replication of H5 and H7 HPAIVs in poultry (2, 16, 50). However, furin-like proprotein convertase subtilisin-kexin (PC/PCSK) genes are a large family that vary with respect to secretion versus transmembrane residence and intracellular localization, as well as the ability to cleave HA (51). It is therefore possible that there are circumstances in which PLG recruitment might be advantageous even in an HPAIV background. Given that PLG recruitment is well correlated with neurovirulence (18) and that several of the recent HPAIV isolates with the potential to recruit PLG are Clade 2.3.4.4b viruses isolated from mammals, including cows (Table 1), determining whether this loss of glycosylation in some HPAIV N1 NAs is genuinely functional or mere sequence noise could be important for understanding the evolution of pathogenicity of this newly emergent variant.

Overall, our study reveals that the unusual HA sequence of Ck/Belgium works in concert with NA-recruited PLG to increase HA cleavage, virus spread, and systemic replication in avian cells. We also provide evidence from another outbreak-associated LPAI H6N1 virus that this is potentially a generalizable virulence mechanism for LPAIVs. We suggest that future outbreaks of unexpectedly pathogenic LPAIVs with an N1 NA in poultry should have their NA and HA genes monitored for loss of the glycosylation site and, secondarily, an atypical HA cleavage sequence.

## SUPPLEMENTAL MATERIAL

Supplemental figures mbio.02466-25-s0001.docx Fig. S1 to S3.

Table S1 mbio.02466-25-s0002.xlsx Genes used in the Fluidigm array.

Table S2 mbio.02466-25-s0003.xlsx Non-laboratory adapted viruses with NAs containing a PLG-binding motif.

Table S3 mbio.02466-25-s0004.xlsx GISAID accession codes used for the analysis described in Fig. 7 and Table S2.

ASM does not own the copyrights to Supplemental Material that may be linked to, or accessed through, an article. The authors have granted ASM a non-exclusive, world-wide license to publish the Supplemental Material files. Please contact the corresponding author directly for reuse.
