# Supplementary material for: Synergy between HA cleavage site sequence and NA-mediated plasminogen recruitment as a virulence mechanism for low-pathogenic avian influenza
Source: mBio. 2026 Feb 26;17(4):e02466-25. doi: 10.1128/mbio.02466-25 (PMC13059709; doi:10.1128/mbio.02466-25)
Supplement: Supplemental figures — Fig. S1 to S3. [file mbio.02466-25-s0001.docx]

**Lee et al, supplementary information**


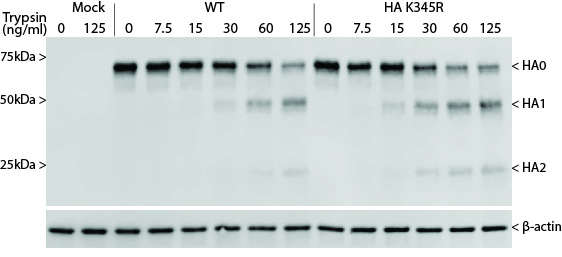


**Figure S1. Trypsin-sensitivity of Ck/Belgium HA in transfected cells.** CLEC213 cells were transfected with plasmids encoding WT or K345R HA for 48 hours and the indicated trypsin concentrations were added for the last 2 hours. SDS-PAGE and western blotting were performed to detect HA and ß-actin. Positions of molecular mass markers are shown on the left.


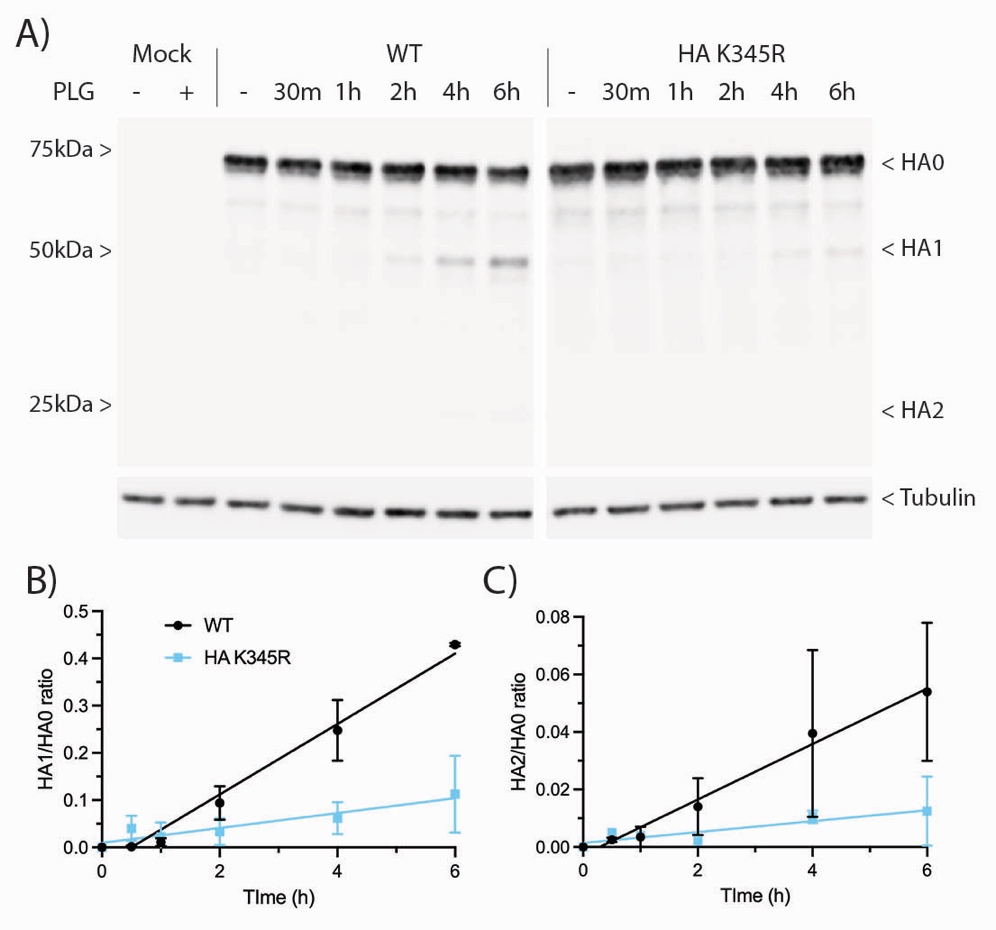


**Figure S2. HA cleavage kinetics of Ck/Belgium HA.** (A-C) CLEC213 cells were infected with WT or HA K345R virus at MOI of 3 or mock infected, treated with 50 μg/ml cycloheximide at 14 hours post-infection, then incubated with 50 ng/ml chicken PLG for 30 minutes, 1, 2, 4 or 6 hours. (A) Cell lysates were examined by SDS-PAGE and western blotting for HA and tubulin. Positions of molecular mass markers are shown on the left. (B, C) Ratios of cleaved to uncleaved HA products from replicate blots were quantified by densitometry. Data points are mean ± SEM of 2 independent experiments fitted to simple linear regression models. HA1/HA0 slopes (arbitrary units) were 0.075 ± 0.01 and 0.015 ± 0.01 for WT and HA K345R respectively. HA2/HA0 slopes were 0.0097 ± 0.004 and 0.0019 ± 0.001.


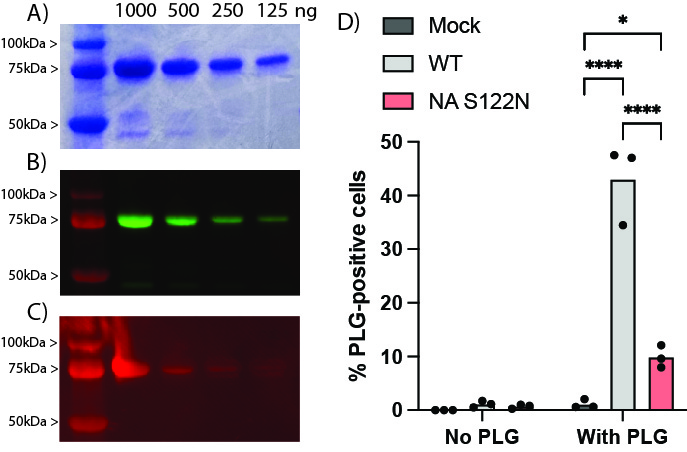


**Figure S3. NA S122N-dependent binding of PLG.** (A-C) Purified chicken PLG was conjugated with AF488 and the indicated amounts were separated by SDS-PAGE and visualised by (A) staining with Coomassie Blue, or (B) imaging of Licor channel 488 or (C) staining with plasminogen polyclonal antibody after western blotting. Positions of molecular mass markers are shown on the left. (D) Cells were infected (or mock infected) at MOI 3 with WT or NA S122N virus and at 14 hours post-infection, treated with or without 50 ng/ml AF488-PLG for 2 hours. Cells were then fixed and the percentage of PLG-positive cells was measured by flow cytometry. Dots on graphs represent percentage of PLG-positive cells from individual experiments and bars indicate the means of 3 independent experiments. Two-way ANOVA followed by Tukey’s multiple comparison test were performed for statistical analyses. * = p < 0.05, **** = p < 0.0001.
